# Supplementary material for: Revisiting the Thickness of the Air–Water Interface from Two Extremes of Interface Hydrogen Bond Dynamics
Source: J Chem Theory Comput. 2024 Oct 4;20(20):9107–15. doi: 10.1021/acs.jctc.4c00457 (PMC11500428; doi:10.1021/acs.jctc.4c00457)
Supplement: Supplementary file 1 — ct4c00457_si_001.pdf [file ct4c00457_si_001.pdf]

Supporting Information for Revisiting the Thickness of the Air-Water Interface from Two Extremes of Interface Hydrogen Bond Dynamics

Gang Huang[†] and Jie Huang[*,‡]

† *Institute of Theoretical Physics, Chinese Academy of Sciences, Zhongguancun East Road 55, 100190 Beijing, China*

‡ *Department of Applied Physics, Aalto University, Helsinki FI-00076, Finland*

E-mail: jie.huang@aalto.fi

1 Hydrogen bond correlation functions

Using a geometric criterion of HB, Luzar and Chandler[1] have pioneered the analysis of HB dynamics of pure water, and subsequently, such analysis has been extended to more complex systems, e.g., electrolytes,[2] protein,[3] and micellar surfaces.[4]

In this work, we mainly used the acceptor-donor-hydrogen (ADH) criterion: two water molecules are H-bonded if their inter-oxygen distance r_{OO} is less than the cutoff radius[5] $r^{\mathrm{c}}_{\mathrm{OO}} = 3.5$ (Å) and the H-O$\cdots$O angle ϕ is less than cutoff angle $\phi^{\mathrm{c}} = 30°$.[6–9] For comparison, we also used another definition of HB (AHD): when the distance between the O atoms of two water molecules is less than the cutoff radius $r^{\mathrm{c}}_{\mathrm{OO}}$, and the O-H$\cdots$O included angle θ is greater than cutoff angle $\theta^{\mathrm{c}} = 120°$, then we say that there is a HB between the two molecules.

We use a configuration $r(t)$ to denote the positions of all the atoms in the system at time t. Either of the criteria above allows one to define an HB population $h[r(t)] = h(t)$, which equals 1 when a tagged pair of molecules are H-bonded, and 0 otherwise. The fluctuation in $h(t)$ from its time-independent equilibrium average is defined by[10] $\delta h = h(t) - \langle h \rangle$. The probability that a specific tagged pair of molecules is H-bonded in a large system is extremely small[1], then $\delta h(t) = h(t)$. Therefore, the correlation of $\delta h(t)$ can be written as

$$\langle \delta h(0) \delta h(t) \rangle = \langle h(0) h(t) \rangle,$$

where the averaging $\langle \cdots \rangle$ is to be performed over the ensemble of initial conditions.

The correlation function $c(t) = \langle h(0)h(t) \rangle / \langle h \rangle$ describes the structural relaxation of H-bonds.[2,11] Here the average $\langle h \rangle$ of the HB population is the probability that a pair of randomly chosen water molecules in the system is H-bonded at any time t. The function $c(t)$ measures correlation in $h(t)$ independent of any possible bond-breaking events, and it relaxes to zero when t is large.[12]

Because the thermal motion can cause distortions of H-bonds from the perfectly tetrahedral configuration, water molecules show a librational motion on a time scale of ~ 0.1 ps superimposed to rotational and diffusional motions (> 1 ps), which causes a time variation in interaction parameters. A new HB population $h^{(\mathrm{d})}(t)$ was also defined to obviate the distortion of real HB dynamics due to the above geometric definition.[2,5] It is 1 when the inter-oxygen distance of a particular tagged pair of water molecules is less than $r^{\mathrm{c}}_{\mathrm{OO}}$ at time t, and 0 otherwise. The H-bonds between a tagged molecular pair that satisfy the condition $h^{(\mathrm{d})}(t) = 1$ may have been broken, but they may more easily form H-bonds again. The correlation function

$$n(t) = \frac{\langle h(0)[1 - h(t)] h^{(\mathrm{d})}(t) \rangle}{\langle h \rangle} \tag{1}$$

[1]The average number of H-bonds in an equilibrium of N water molecules is: $\frac{1}{2}N(N-1)\langle h \rangle \sim N$. It implies that $\frac{1}{2}N\langle h \rangle \sim 1$. For a large system, $\langle h \rangle$ is extremely small.

represents the probability at time t that a tagged pair of initially H-bonded water molecules are unbonded but remain separated by less than $r_{\mathrm{OO}}^{\mathrm{c}}$.[2]

The rate of HB relaxation to equilibrium is characterized by the reactive flux[13]

$$k(t) = -\frac{\mathrm{d}c(t)}{\mathrm{d}t}, \tag{2}$$

which quantifies the rate that an initially present HB breaks at time t, independent of possible breaking and reforming events in the interval from 0 to t. Therefore, $k(t)$ measures the effective decay rate of an the initial set of H-bonds.[10] For bulk water, a 0.2-ps transient period exists, during which $k(t)$ changes quickly from its initial value.[14] However, at longer times, $k(t)$ is independent of the HB definitions.

2 Hydrogen bond breaking and reforming rate constants

Assume that each HB acts independently of other H-bonds,[1,13] and due to the detailed balance condition, one obtains $\tau_{\mathrm{HB}} = (1 - \langle h \rangle)/k$, where k is the rate constant of breaking an HB, i.e., the forward rate constant.[15,16] Correspondingly, the backward rate constant k' is represented by the rate constant from the HB-on state to the HB-off state for a tagged pair of molecules. Based on the functions $n(t)$, $h(t)$, $h^{(\mathrm{d})}(t)$, and $k(t)$, Khaliullin and Kühne[17] have obtained the ratio k/k' of HB breaking and reforming rate constants in bulk water, and then the lifetime and relaxation time of H-bonds from simulations. Here, for the air-water interface, we obtain the optimal solution range of k and k' from the relationship between the reactive flux $k(t)$ and the correlation functions $c(t)$ and $n(t)$:

$$k(t) = kc(t) - k'n(t). \tag{3}$$

We obtain the optimal value of the rate constants, k and k', by the least squares fit of $k(t)$, $c(t)$, and $n(t)$ beyond the transition phase. The function $c(t)$ is regarded as a column vector composed of $(c_1, \cdots, c_P)^{\mathrm{T}}$, and is denoted as $\mathbf{c}$, with c_i representing the value of correlation $c(t)$ at $t = i$. Similarly, $n(t)$ and $k(t)$ can also be denoted as $\mathbf{n}$ and $\mathbf{k}$, respectively. Then, the rate constants k and k' are determined from the matrix $\mathbf{A} = \begin{bmatrix} \mathbf{c} & \mathbf{n} \end{bmatrix}$:

$$\begin{bmatrix} k \\ -k' \end{bmatrix} = (\mathbf{A}^{\mathrm{T}}\mathbf{A})^{-1}\mathbf{A}^{\mathrm{T}}\mathbf{k}. \tag{4}$$

3 Details of Scenario 1: the Luzar-Chandler (LC) method

In Scenario 1, to obtain interface HB dynamics, we use molecule sampling at the instantaneous interface. Let T_{e} be the time it takes for all water molecules in the simulation box to traverse the interface and bulk phase, and τ_{HB} be the characteristic time of HB dynamics.

If the trajectory length t_{traj} satisfies the condition $\tau_{\mathrm{HB}} \ll t_{\mathrm{traj}} \ll T_{\mathrm{e}}$, we sample interfacial water molecules at time points t_p $(p = 1, \cdots, M)$ that are evenly spaced on the trajectory, then obtain interface HB dynamics using the following procedure:

1. For each simulation time step, define a pair of interfaces with a thickness d as shown in Figure 1 in the main text. At each t_p, we select a set S_p of water molecules in the pair of interfacial layers $\mathcal{I}(d;t)$, i.e., $\mathbf{r}(t_p) \in \mathcal{I}(d;t_p)$. The set of interfacial water molecules is given by the union of S_p. This sampling process of interface water can be seen in Figure S1. Then we calculate the correlation functions $c(t)$, $n(t)$, and $k(t)$ through t_{traj} for these water molecules belonging to the set S_p of interfacial water molecules.

2. Calculate average functions of the correlation functions $c(t)$, $n(t)$, and $k(t)$ respectively over all sub-trajectories.

3. Calculate reaction rate constants of breaking and reforming for the interfacial layer $\mathcal{I}(d;t)$ by Equation 3.

In Scenario 1, the interfacial water-picking is performed every 4 ps.

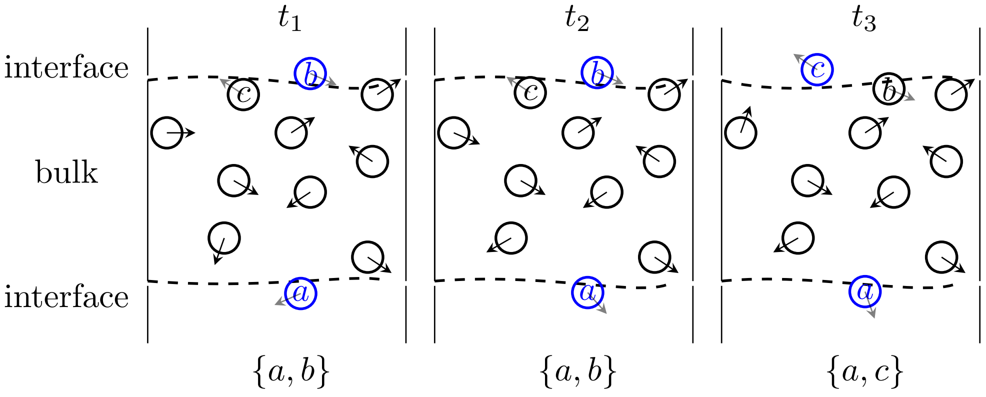

Figure S1: The sampling of water molecules at the interface in Scenario 1. The figure shows the configuration of the system at three water-picking moments. As a schematic diagram, we use circles to represent water molecules and dotted curves to represent the boundaries of the air-water interface. The three moments t_1, t_2, and t_3 belong to the same sub-trajectory. At these three moments, the interface water molecules (in blue) we selected are $\{a, b\}$, $\{a, b\}$ and $\{a, c\}$, respectively. Therefore, in this sub-trajectory, we use their union as the interface water molecules, that is, $\{a, b, c\}$.

4 Rotational anisotropy decay of OH stretch at the air-water interface

We examine the water interface thickness results from the interfacial HB dynamics by polarization anisotropy decay of OH stretch. The polarization anisotropy decay of the OH stretch can also provide information on the water dynamics .[18–21] We assume that the anisotropy decay $R(t)$ is only due to the orientational relaxation of water molecules. Within the Condon approximation ,[22] it is directly related to the orientational correlation function $C_2(t)$[21,23–25] through $R(t) = \frac{2}{5}C_2(t)$. The $C_2(t)$ is given by the rotational time-correlation function

$$C_2(t) = \langle P_2(\hat{u}(0) \cdot \hat{u}(t)) \rangle, \tag{5}$$

where $P_2(x)$ is the second-order Legendre polynomial ,[26] i.e., $P_2(x) = \frac{1}{2}(3x^2 - 1)$, $\hat{u}(t)$ is the time-dependent unit vector of the transition dipole, and $\langle\rangle$ indicate equilibrium ensemble

average .[27] In our simulations, we concentrate on water molecules and consider a unit vector directed along the OH bond.

For the air-water interface, using the Method section in the main text, we obtain the instantaneous interface with thickness $d = 1, \cdots, 6$ (Å). Then, for each value of d, the LC method is used to obtain $C_2(t)$ for water molecules at the air-water interface.

5 Computational Methods

5.1 Density functional theory-based molecular dynamics (DFTMD) simulations

To describe the subtleties of H-bonding in water,[28] we have performed DFTMD simulations[29] for bulk water and the air-water interface. The simulations make use of technologies that have been successfully tested on water and solutions,[30–32] namely the Goedecker-Teter-Hutter (GTH) pseudopotentials,[33–35] Generalised Gradient Approximation (GGA) of the exchange-correlation functional,[36,37] and dispersion force correction, DFT-D3.[38,39] By eliminating the strongly bound core electrons, the GTH pseudopotentials reduce the number of occupied electronic orbitals that have to be treated in an electronic structure calculation. There are dual-space Gaussian-type pseudopotentials that are separable and satisfy a quadratic scaling to system size.[40] The GGA functionals generally describe the dipole and quadruple moments of the molecules quite well, and the DFT-D3 correction treats the van der Waals dispersion forces and improves the structural properties without more computational cost and thus can be used for the air-water interface.

The DFTMD calculation is implemented by an NVT code that is implemented in the CP2K/QUICKSTEP package.[41,42] The BLYP XC functional, which consists of Becke non-local exchange[36] and Lee-Yang-Parr correlation[37] has been employed. The electron-ion interactions are described by GTH pseudopotentials.[34,43] A Gaussian basis for the wave functions and an auxiliary plane wave basis set for the density is used in this scheme. The DZVP-GTH

basis set is used for all atoms and a cutoff of 280 (Ry) is chosen for the charge density.[41] The Nosé-Hoover chain thermostat[44] is used to conserve the temperature at 300 (K). The simulation for the air-water interface uses a time step of 0.5 (fs). The reference density for determining the instantaneous interface is set to 0.016 (g/cm^3).

The bulk water system consisted of 128 water molecules in a periodic box of size $15.64 \times 15.64 \times 15.64$ (Å^3), and with a density of 1.00 (g/cm^3). The slab consisted of 128 water molecules in a periodic box of size $15.64 \times 15.64 \times 31.28$ (Å^3). The length of each trajectory is 60 (ps).

5.2 Deep potential molecular dynamics (DeePMD) simulations

DeePMD simulations based on MB-pol[45–48] were conducted using LAMMPS[49] package for different sizes of air-water interfaces, ranging from 125 to 1000 water molecules. Initially, water molecules were placed in the middle of a simulation box in 3D periodic boundary conditions. The equations of motion were integrated using the velocity-Verlet algorithm with a time step of 0.5 (fs). The temperature was maintained at 300 (K) using a Nosé–Hoover thermostat with a time constant of 0.5 (ps). The system was simulated in the NVT ensemble, ensuring the constant number of particles, volume, and temperature throughout the simulation. Details of simulation settings for all systems are provided in Table S1.

Table S1: Number (N) of water molecules, box dimensions (a, b, c) along x, y and z axes, temperature (Temp.) and surface tension γ values for different air-water interface systems.

N	125	216	343	512	729	1000
a (Å)	15.52	19.71	21.72	24.63	27.93	31.28
b (Å)	15.52	19.71	21.72	24.63	27.93	31.28
c (Å)	77.60	98.55	108.64	123.19	139.69	156.44
Temp. (K)	300	300	300	300	300	300
Time (ps)	842	775	300	705	300	300
γ (mN/m)	68.93±3.48	71.53±3.80	70.23±3.48	69.60±2.60	70.72±4.10	70.09±4.96

5.3 Surface tension calculations

The surface tension γ is calculated from the pressure tensor by considering all the atoms in a system:

$$\gamma = \frac{L_z}{2}\left[\langle P_{zz}\rangle - 0.5\left(\langle P_{xx}\rangle + \langle P_{yy}\rangle\right)\right] \tag{6}$$

where L_z is the box length in the z-dimension, and P_{ii} are the diagonal components of the pressure tensor. The factor 2 accounts for the two liquid-vapor interfaces in the system.[50–52] In practice, the pressure tensors are obtained from the thermodynamic log from LAMMPS.[49] For each non-overlapping 40 ps sub-trajectory along the entire trajectory, we conduct one measurement of surface tension through Equation 6. The sliding value of surface tension is then calculated across the whole trajectory. As shown in Figure S2, both sliding and accumulated surface tension values are plotted.

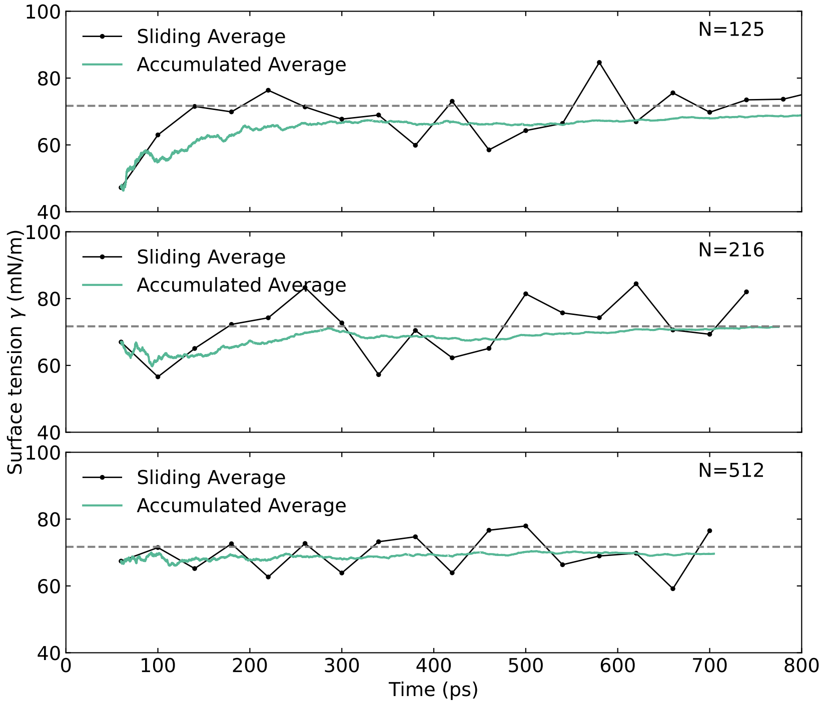

Figure S2: Time evolution of the sliding and accumulated surface tension values as computed by DeePMD simulations. The time length of a sub-trajectory used for one sliding average is 40 ps.

The surface tension value for each system is estimated from the sliding values, as detailed in Table S1 and Figure S11. It is worth noting that a sufficiently long simulation time is needed to obtain a stable surface tension value. The calculated surface tension values are consistent with the experimental value (71.70 mN/m).[53] Although long-range corrections are crucial for precise surface tension calculations, they were not included in our study due to its specific focus. These corrections are particularly important when precise quantification of surface tension is necessary.[54,55]

6 Results for verification

6.1 Results based on DFTMD simulations

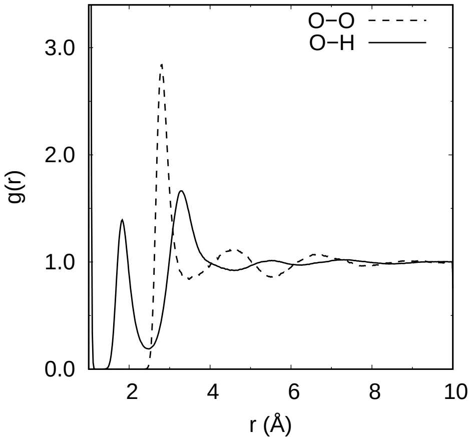

Figure S3: The partial radial distribution functions (RDFs) for the simulated bulk water.

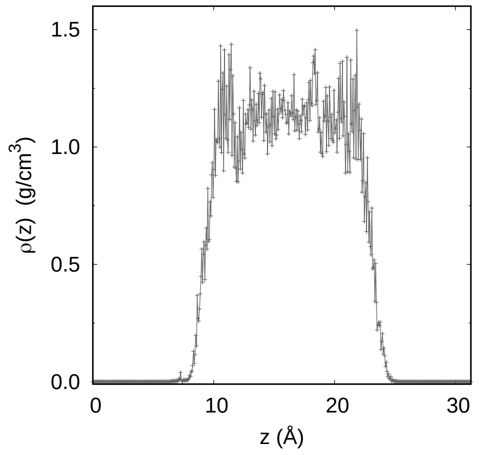

Figure S4: The density $\rho(z)$ along the interface normal of the slabs of 128 water molecules, simulated by DFTMD simulations. It can be seen that in the middle part of the interface, the density of water is 1.0 g/cm^3. This value is close to the density of the bulk water.

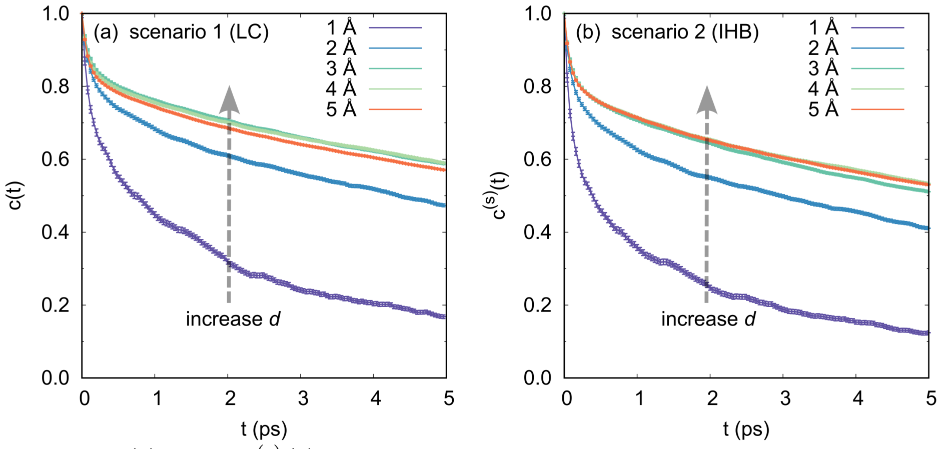

Figure S5: The $c(t)$ and $c^{(\mathrm{s})}(t)$ for interface H-bonds obtained from the ADH criterion $(r_{\mathrm{OO}}^{\mathrm{c}}, \phi^{\mathrm{c}}) = (3.5\ \text{Å}, 30^{\circ})$ of HB definition for (a) Scenario 1 (LC); (b) Scenario 2 (IHB). Two features can be found: (i). As d increases, both $c(t)$ and $c^{(\mathrm{s})}(t)$ eventually approach a stable function. (ii). The decay rate of $c^{(\mathrm{s})}(t)$ in Scenario 2 surpasses $c(t)$ in Scenario 1. This is visually represented by two dashed directed line segments, positioned identically to the graphs.

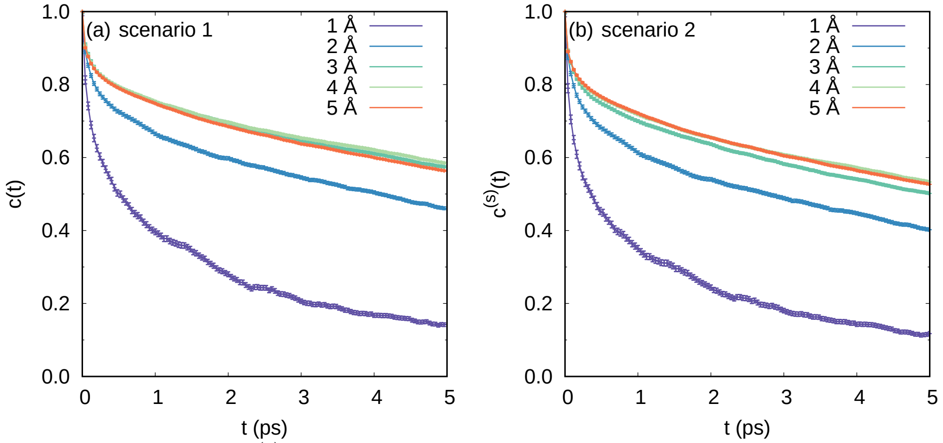

Figure S6: The $c(t)$ and $c^{(\mathrm{s})}(t)$ for interface H-bonds obtained from the AHD criterion $(r^{\mathrm{c}}_{\mathrm{OO}}, \theta^{\mathrm{c}}) = (3.5\ \text{Å}, 120°)$ of HB definition for (a) Scenario 1 (LC); (b) Scenario 2 (IHB). Similar to the case of ADH, the same features can be found: (i). As d increases, both $c(t)$ and $c^{(\mathrm{s})}(t)$ eventually approach a stable function. (ii). The decay rate of $c^{(\mathrm{s})}(t)$ in Scenario 2 surpasses $c(t)$ in Scenario 1. As can be seen from Figures S5 and S6, the above two features do not particularly depend on the specific definition of H-bonds.

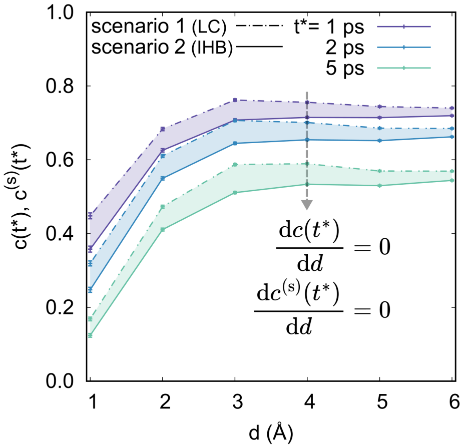

Figure S7: The d-dependence of the correlation functions at reference time points $t^* = 1, 2, 5$ (ps), revealing key insights into the dynamics of the air-water interface: (i). As d increases, both $c(t^*)$ and $c^{(\mathrm{s})}(t^*)$ show an upward trend, with their rates of change gradually approaching 0. (ii). For each t^*, $c(t)$ is slightly larger than $c^{(\mathrm{s})}(t)$ for the same d. (iii). The thickness $d_f = 4$ (Å) of the air-water interface is obtained from the d-dependence of $c(t^*)$ and $c^{(\mathrm{s})}(t^*)$, obtained by the LC method (dot-dashed lines) and the IHB method (solid lines), respectively.

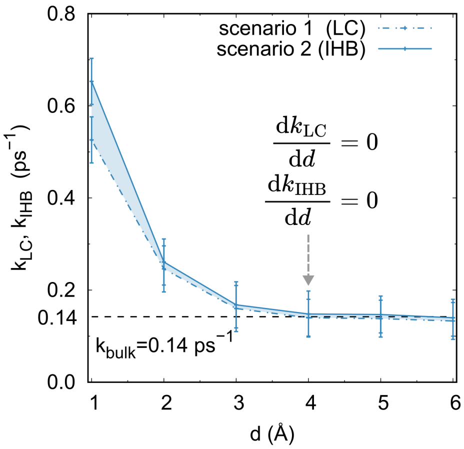

Figure S8: The breaking HB reaction rate constants $k_{\rm LC}$ and $k_{\rm IHB}$ obtained by the IHB method and the LC method, respectively. (i). Both constants, $k_{\rm LC}$ and $k_{\rm IHB}$, decrease monotonically to the HB breaking rate $k_{\rm bulk}$ for the bulk water as d increases. (ii). $k_{\rm LC}$ is smaller than $k_{\rm IHB}$ for the same d. (iii). The thickness $d'_f = 4$ (Å) of the air-water interface is obtained from the d-dependence of $k_{\rm IHB}$ and $k_{\rm LC}$, respectively.

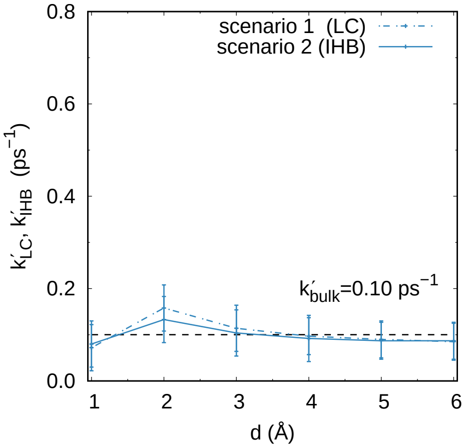

Figure S9: The d-dependence of the rate constant $k'_{\rm IHB}$ and $k'_{\rm LC}$, obtained by the IHB (solid lines) and the Luzar-Chandler (dot-dashed lines) methods, respectively. The corresponding reaction rate $k'_{\rm bulk}$ for bulk water is also represented as dashed lines. The ADH criterion of H-bonds is used and the fits are carried on the time region $0.2 < t < 12$ (ps).

For bulk water and the air-water interface, the k and k' are reported in Table S2 and S3.

Table S2: The k and k' for the bulk water and the air-water interface (the time region $0.2 < t < 2$ (ps)).

Criterion	k (b)[2]	k' (b)	$\tau_{\rm HB}$ (b)[3]	k (i)	k' (i)	$\tau_{\rm HB}$ (i)
ADH	0.296	0.988	3.380	0.323	0.765	3.101
AHD	0.288	1.149	3.470	0.314	0.887	3.184

Table S3: The k and k' for the bulk water and the air-water interface (the time region $2 < t < 12$ (ps)).

Criterion	k (b)	k' (b)	$\tau_{\rm HB}$ (b)	k (i)	k' (i)	$\tau_{\rm HB}$ (i)
ADH	0.115	0.039	8.718	0.157	0.068	6.372
AHD	0.105	0.047	9.496	0.155	0.088	6.472

6.2 Results based on DeePMD simulations

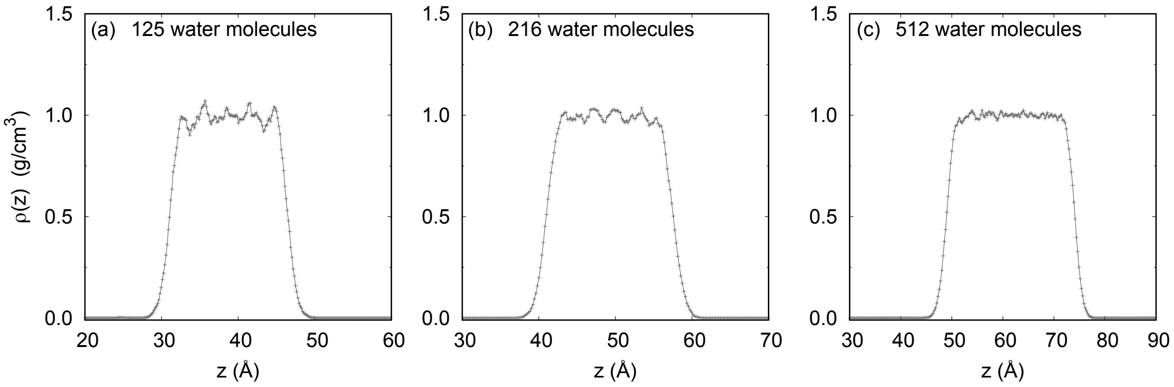

Figure S10: The Density $\rho(z)$ along the interface normal of H_2O in the slabs of 125 (a), 216 (b), and 512 (c) water molecules, simulated by DeePMD simulations with MB-pol. The density values for these systems closely align well with the bulk water density, 1.0 (g/cm^3).

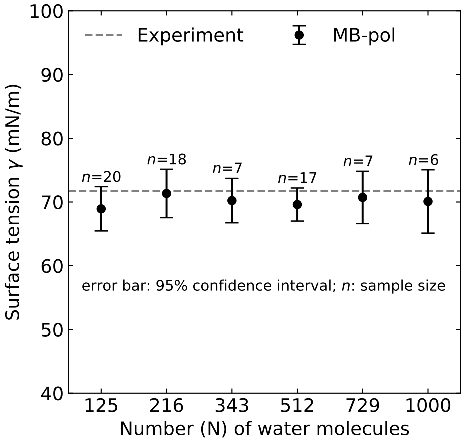

Figure S11: Surface tension γ for different size of systems. The surface tension values are consistent with the experimental value of 71.70 (mN/m) at 300 (K).[53] The error bars represent the 95% confidence interval, and sample size n, i.e., the number of non-overlapped 40 ps sub-trajectories, to calculate the error bar are shown in the figure.

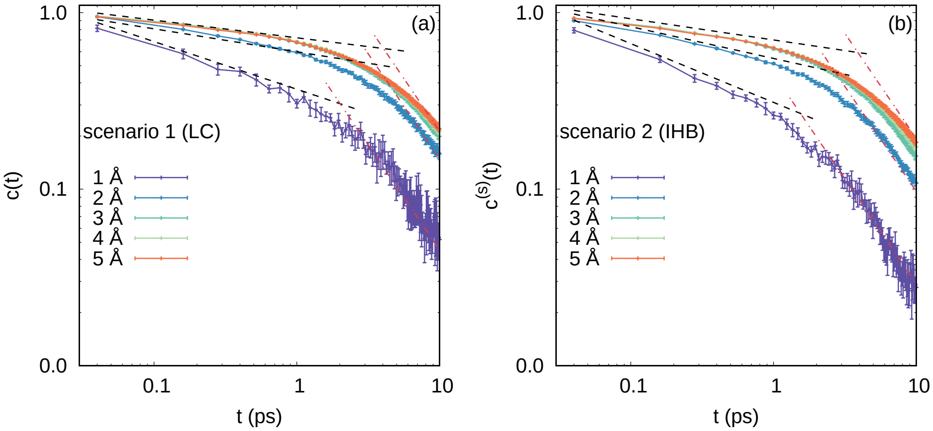

Figure S12: The auto-correlation functions for interface H-bonds with varying thickness d are displayed on a log-log scale for (a) Scenario 1 (LC) and (b) Scenario 2 (IHB). With a critical time t^{c} serving as the boundary, the decay of $c(t)$ and $c^{(s)}(t)$ can be characterized by two power-law phases: short-term decay and long-term decay. In the short-term phase, both $c(t)$ and $c^{(s)}(t)$ follow a $t^{-\alpha_1}$ law, with the exponent α_1 decreasing from 0.3 to 0.1 as d increases from 1 to 5 (Å). The long-term decay follows a $t^{-\alpha_2}$ law with an exponent $\alpha_2 \approx 1.2$ for all thicknesses. For both scenarios, $t^{c} \approx 2$ (ps) for $d = 1$ (Å), and t^{c} increases with the increase of d.

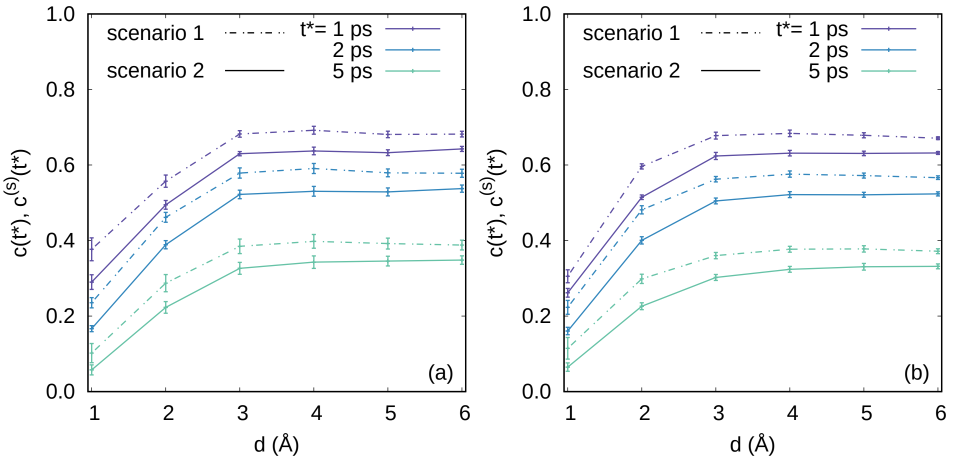

Figure S13: The d-dependence of the correlation function $c(t^*)$ at three reference time points $t^* = 1, 2, 5$ (ps), revealing key insights into the dynamics of two air-water interface models, simulated by DeePMD with MB-pol: (a) 125 water molecules; (b) 216 water molecules. Similar to the results of the interface model of 512 water molecules. (i). As d increases, both $c(t^*)$ and $c^{(\mathrm{s})}(t^*)$ show an upward trend, with their rates of change gradually approaching 0. (ii). For each t^*, $c(t)$ is slightly larger than $c^{(\mathrm{s})}(t)$ for the same d. (iii). The thickness $d_f = 4$ (Å) of the air-waterinterface is obtained from the d-dependence of $c(t^*)$ and $c^{(\mathrm{s})}(t^*)$, obtained by the LC method (dot-dashed lines) and the IHB method (solid lines).

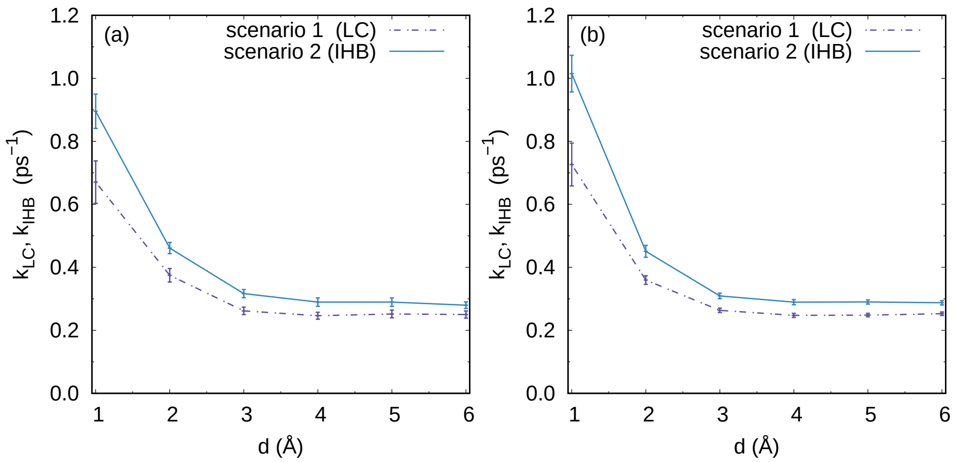

Figure S14: The breaking HB reaction rate constants k_{LC} and k_{IHB} obtained by the LC and IHB methods respectively: (a) 125 water molecules; (b) 216 water molecules.

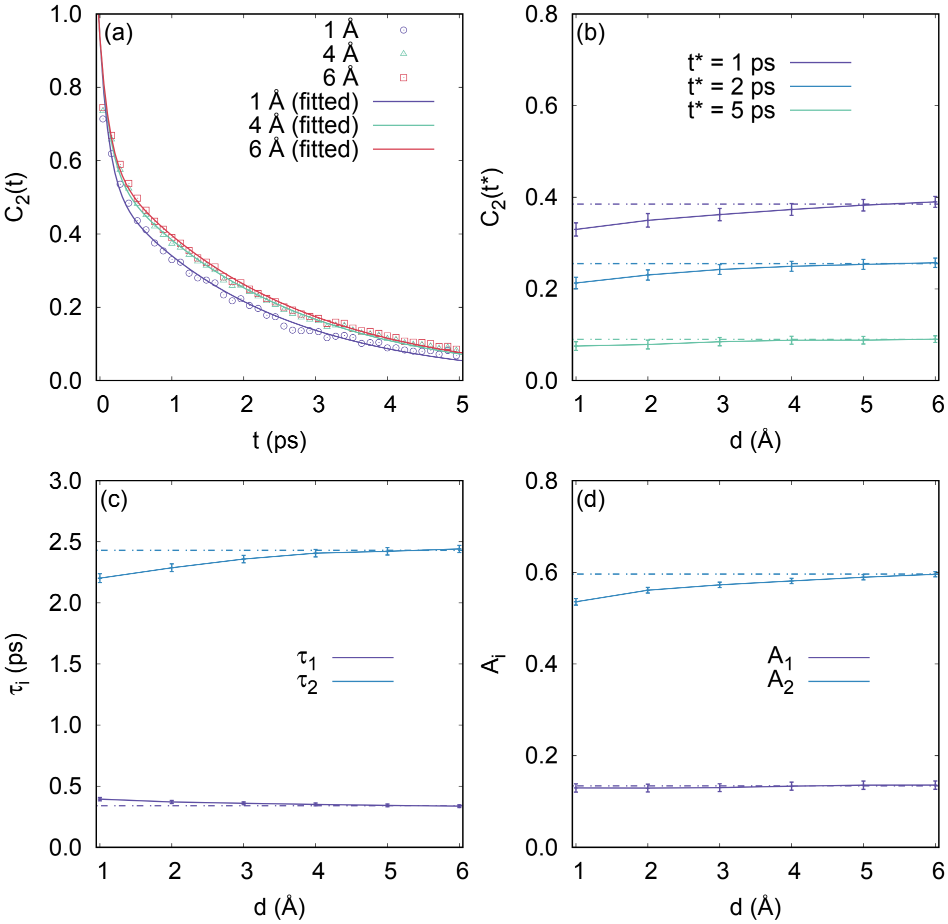

Figure S15: The orientation correlation function $C_2(t)$ for the air-water interface simulated with 125 water molecules. (a) The $C_2(t)$ for $d = 1, 4, 6$ (Å) and the bi-exponential fitted functions $C_2(t) = A_1 \exp(t/\tau_1) + A_2 \exp(t/\tau_2)$. The d-dependence of (b) $C_2(t^*)$ for water molecules at the air-water interface with $t^* = 1, 2, 5$ (ps) and (c) relaxation time (τ_1 and τ_2), and (d) the amplitude (A_1 and A_2). The dotted lines are horizontal to enhance visibility (this applies to Figure S16 as well).

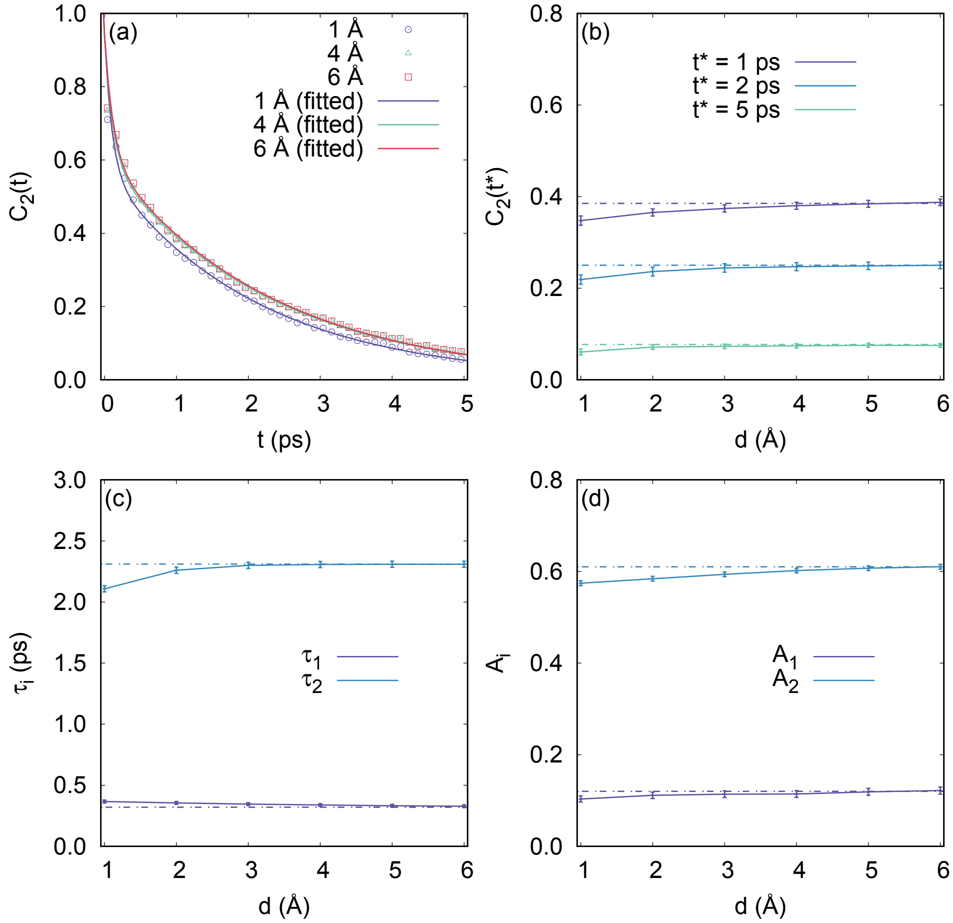

Figure S16: The orientation correlation function $C_2(t)$ for the air-water interface simulated with 216 water molecules. (a) The $C_2(t)$ for $d = 1, 4, 6$ (Å) and the bi-exponential fitted functions $C_2(t) = A_1 \exp(t/\tau_1) + A_2 \exp(t/\tau_2)$. The d-dependence of (b) $C_2(t^*)$ for water molecules at the air-water interface with $t^* = 1, 2, 5$ (ps) and (c) relaxation time (τ_1 and τ_2), and (d) the amplitude A_1 and A_2. From the convergence trend of τ_1 and τ_2 in (c), we find that the OH orientation relaxation of the air-water interface no longer changes at the interface with a thickness greater than 4 (Å). From Figure S15 and this figure, as d increases to 4 (Å), $C_2(t^*)$ and τ_i, A_i all converge to a fixed value, respectively. The results are consistent with the results of Figure 5 and with the conclusion that the air-water interface thickness is 4 (Å) given by the interfacial HB dynamics.

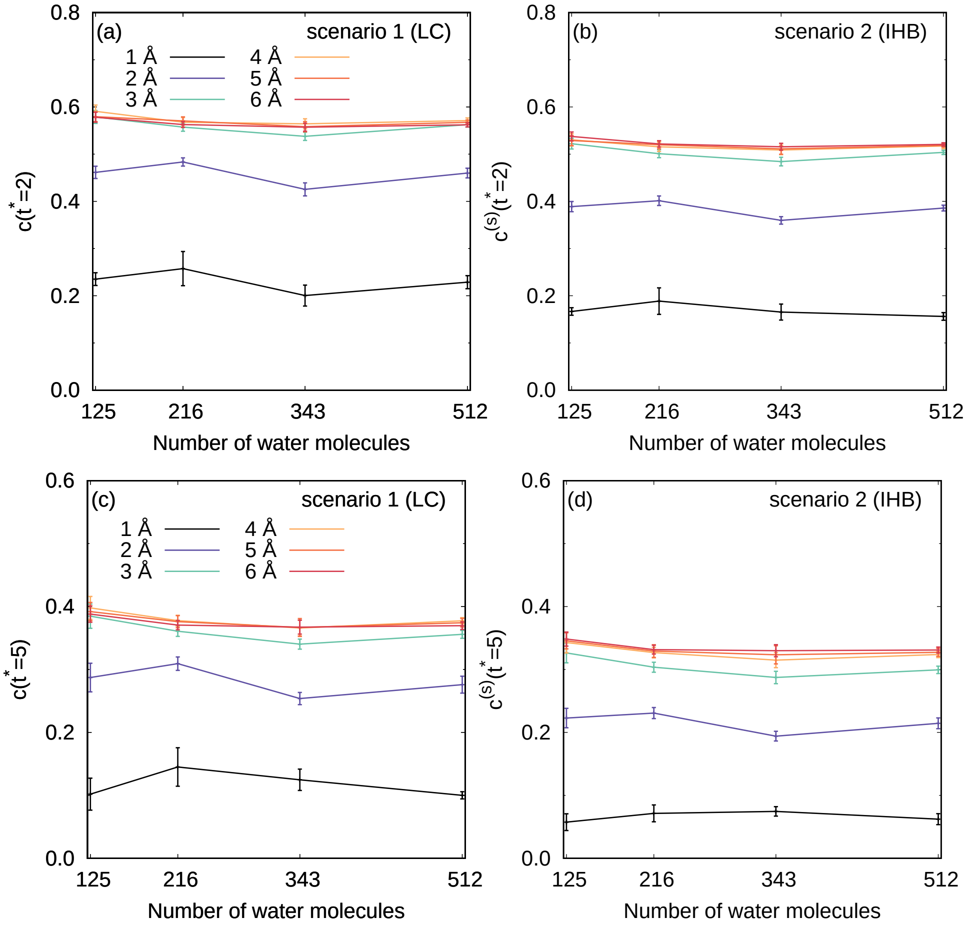

Figure S17: The system size-dependence of HB population correlation functions for the air-water interface at reference time t^* in two scenarios respectively: (a–b) $t^* = 2$ (ps); (c–d) $t^* = 5$ (ps). For small d, $c(t^*)$ ($c^{(\mathrm{s})}(t^*)$) fluctuates slightly with the number (N) of molecules, and its standard error gradually decreases. For $d \geq 4$ (Å), their values do not dependent on N when $N \geq 216$.

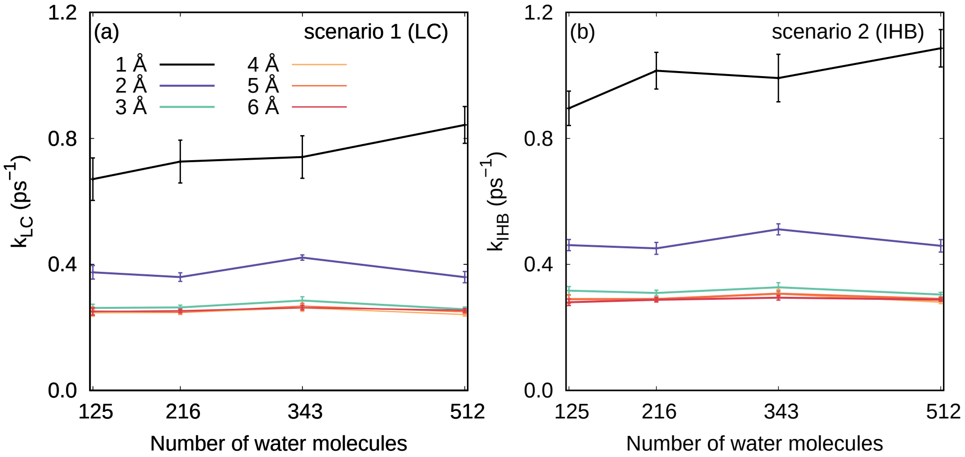

Figure S18: The system size-dependence of the (a) k_{LC} and (b) k_{IHB} for the air-water interface. There are fluctuations when $d \leq 2$ (Å), and there is a slight trend of increasing with the increase of N. However, when $d \geq 4$ (Å), the N-dependence of the reaction constant is not obvious.

6.3 Perspectives Derived from the Correlation Function of Free OH Groups

Similar to the interfacial HB dynamics, we also determine the thickness of the air-water interface from the perspective of free OH groups. Based on the definition of the free OH group defined in Reference 56, we define *interfacial* free OH group population $n_{\mathrm{f}}^{(\mathrm{s})}$ at the air-water interface as

$$n_{\mathrm{f}}^{(\mathrm{s})}[\mathbf{r}(t)] = \begin{cases} 1 & \mathbf{r}_i \in \mathcal{I}(d;t), \\ & i \text{ is not H-bonded;} \\ 0 & \text{otherwise} \end{cases} \tag{7}$$

where $\mathbf{r}(t)$ is the configuration of the system at time t, $\mathbf{r}_i$ is the position coordinate of the oxygen atom in an OH group, and $\mathcal{I}(d;t)$ is the instantaneous interface layer with thickness d at time t. To test our previous results further, the auto-correlation function

$$c_{\mathrm{f}}^{(\mathrm{s})}(t) = \frac{\langle n_{\mathrm{f}}^{(\mathrm{s})}(0) n_{\mathrm{f}}^{(\mathrm{s})}(t) \rangle}{\langle n_{\mathrm{f}}^{(\mathrm{s})} \rangle} \tag{8}$$

for the interfacial free OH group population based on both R-β and R-θ definition[56] of HB are calculated. In the calculation of this correlation function, the optimized geometry-based definitions for the free OH groups are given by $(R_{\mathrm{c}}, \beta_{\mathrm{c}}) = (3.5\ \text{Å}, 50°)$ and $(R_{\mathrm{c}}, \theta_{\mathrm{c}}) = (3.5\ \text{Å}, 110°)$, respectively.[56] The correlation for the air-water interface based on two definitions of HB are shown in Figure S19a-b, respectively. It can be seen that, with a critical time $t_{\mathrm{c}} \approx 1.7$ (ps) (marked by black arrows in Figure S19a-b) as the boundary, there is a significant difference in the dependence of $c_{\mathrm{f}}^{(\mathrm{s})}(t)$ on the depth d: when $t < t_{\mathrm{c}}$, the larger d is, the faster the relaxation of $c_{\mathrm{f}}^{(\mathrm{s})}(t)$ is; when $t > t_{\mathrm{c}}$, the opposite is true. These results imply that these free OH groups close to the bulk phase appear to be freer than they are on the interface in a short time. However, from a longer time scale, their movement is not as fast as that on the interface. Despite the above differences, whether $t < t_{\mathrm{c}}$ or $t > t_{\mathrm{c}}$, the rate of

change of the correlation function $c_{\mathrm{f}}^{(\mathrm{s})}(t)$ with d is basically equal to 0 when $d = 5$ (Å) This conclusion can be seen from the slope $\mathrm{d}[c_{\mathrm{f}}^{(\mathrm{s})}(t^*)]/\mathrm{d}d$ for different reference time t^* as shown in Figure S19c.

Based on the correlation function $c_{\mathrm{f}}^{(\mathrm{s})}(t^*)$ at reference time $t^* = 1, 2, 5$ (ps), we conclude that the air-water interface thickness $d_f \approx 5$ (Å) is confirmed from the d-dependence of $c_{\mathrm{f}}^{(\mathrm{s})}(t^*)$ in both R-β and R-θ definitions of HB, respectively.

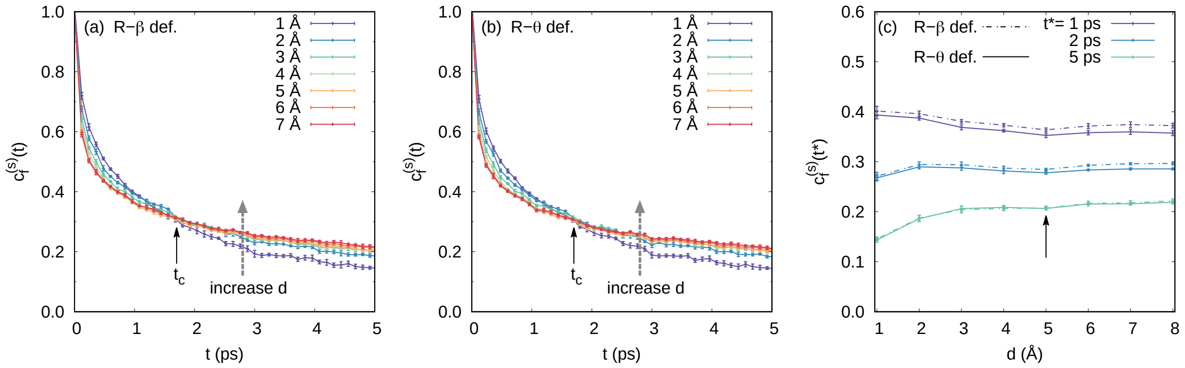

Figure S19: The free OH group correlation functions for the air-water interface simulated with 512 water molecules. The number N_{s} of samples is 6. (a-b)The time-dependence of the correlations $c_{\mathrm{f}}^{(\mathrm{s})}(t)$. The definition of the free OH group is based on the optimized R-β and R-θ definition of HB,[56] respectively. (c) The d-dependence of the correlation function $c_{\mathrm{f}}^{(\mathrm{s})}(t^*)$ at three reference time points $t^* = 1, 2, 5$ (ps). The approximate value of d_f is marked by a black arrow. (Consistent results for systems with 125 and 216 water molecules are shown in Figures S20 and S21.)

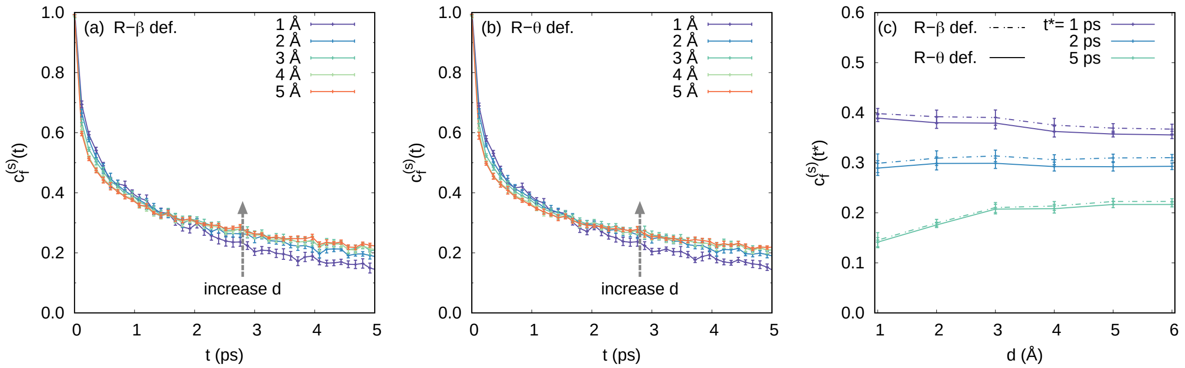

Figure S20: (a-b)The time-dependence of the correlation functions $c_{\mathrm{f}}^{(\mathrm{s})}(t)$ for air-water interface simulated with 125 water molecules. The definition of the free OH group is based on (a) R-β and (b) R-θ definition of HB,[56] respectively. (c) The d-dependence of the correlation function $c_{\mathrm{f}}^{(\mathrm{s})}(t^*)$ at three reference time points $t^* = 1, 2, 5$ (ps).

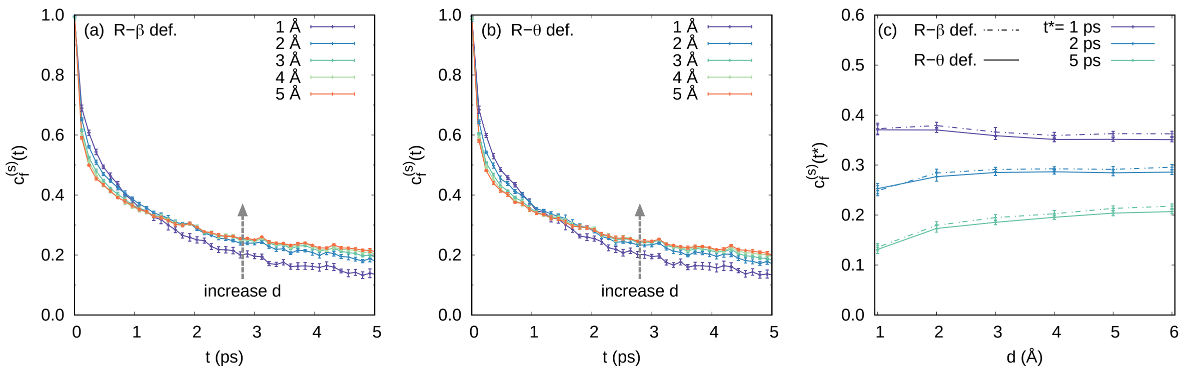

Figure S21: (a-b)The time-dependence of the correlations $c_{\rm f}^{(\rm s)}(t) = \langle n_{\rm f}^{(\rm s)}(0) n_{\rm f}^{(\rm s)}(t)\rangle / \langle n_{\rm f}^{(\rm s)}\rangle$ for air-water interface simulated with 216 water molecules. The definition of the free OH group is the same as Figure S20. (c) The d-dependence of the correlation function $c_{\rm f}^{(\rm s)}(t^*)$ at three reference time points $t^* = 1, 2, 5$ (ps).

References

(1) Luzar, A.; Chandler, D. Effect of Environment on Hydrogen Bond Dynamics in Liquid Water. *Phys. Rev. Lett.* **1996**, *76*, 928–931.

(2) Chandra, A. Effects of Ion Atmosphere on Hydrogen-Bond Dynamics in Aqueous Electrolyte Solutions. *Phys. Rev. Lett.* **2000**, *85*, 768–771.

(3) Tarek, M.; Tobias, D. J. Role of protein-water hydrogen bond dynamics in the protein dynamical transition. *Phys. Rev. Lett.* **2002**, *88*, 138101.

(4) Pal, S.; Bagchi, B.; Balasubramanian, S. Hydration Layer of a Cationic Micelle, C10TAB: Structure, Rigidity, Slow Reorientation, Hydrogen Bond Lifetime, and Solvation Dynamics. *J. Phys. Chem. B* **2005**, *109*, 12879–12890.

(5) Sciortino, F.; Fornili, S. L. Hydrogen Bond Cooperativity in Simulated Water: Time Dependence Analysis of Pair Interactions. *J. Chem. Phys.* **1989**, *90*, 2786–2792.

(6) Soper, A. K.; Phillips, M. G. A New Determination of the Structure of Water at 25 °C. *Chem. Phys* **1986**, *107*, 47–60.

(7) Teixeira, J.; Bellisent-Funel, M. C.; Chen, S. H. Dynamics of Water Studied by Neutron Scattering. *J. Phys. Condens. Matter* **1990**, *2*, SA105.

(8) Luzar, A.; Chandler, D. Structure and hydrogen bond dynamics of water-dimethyl sulfoxide mixtures by computer simulations. *J. Chem. Phys.* **1993**, *98*, 8160–8173.

(9) Balasubramanian, S.; Pal, S.; Bagchi, B. Hydrogen-Bond Dynamics Near a Micellar Surface: Origin of the Universal Slow Relaxation at Complex Aqueous Interfaces. *Phys. Rev. Lett.* **2002**, *89*, 115505.

(10) Chandler, D. *Introduction to Morden Statistical Mechanics*; Oxford University press, Oxford, 1987.

(11) Benjamin, I. Hydrogen Bond Dynamics at Water/Organic Liquid Interfaces. *J. Phys. Chem. B* **2005**, *109*, 13711–13715.

(12) Rapaport, D. C. Hydrogen Bonds in Water: Network Organization and Lifetimes. *Mol. Phys.* **1983**, *50*, 1151–1162.

(13) Luzar, A. Resolving the Hydrogen Bond Dynamics Conundrum. *J. Chem. Phys.* **2000**, *113*, 10663.

(14) Starr, F. W.; Nielsen, J. K.; Sta, H. E. Hydrogen-Bond Dynamics for the Extended Simple Point-Charge Model of Water. *Phys. Rev. E* **2000**, *62*, 579–587.

(15) Chandler, D. Roles of Classical Dynamics and Quantum Dynamics on Activated Processes Occurring in Liquids. *J. Stat. Phys.* **1986**, *42*, 49–67.

(16) Chandler, D. Statistical Mechanics of Isomerization Dynamics in Liquids and the Transition State Approximation. *J. Chem. Phys.* **1978**, *68*, 2959–2970.

(17) Khaliullin, R. Z.; Kühne, T. D. Microscopic Properties of Liquid Water from Combined Ab Initio Molecular Dynamics and Energy Decomposition Studies. *Phys. Chem. Chem. Phys.* **2013**, *15*, 15746–15766.

(18) Woutersen, S.; Bakker, H. J. Resonant intermolecular transfer of vibrational energy in liquid water. *Nature* **1999**, *402*, 1.

(19) Cowan, M. L.; Bruner, B. D.; Huse, N.; Dwyer, J. R.; Chugh, B.; Nibbering, E. T. J.; Elsaesser, T.; Miller, R. J. D. Ultrafast memory loss and energy redistribution in the hydrogen bond network of liquid H_2O. *Nature* **2005**, *434*, 199.

(20) Kraemer, D.; Cowan, M. L.; Paarmann, A.; Huse, N.; Nibbering, E. T. J.; Elsaesser, T.; Miller, R. J. D. Temperature dependence of the two-dimensional infrared spectrum of liquid H2O. *Proc. Natl. Acad. Sci. USA* **2008**, *105*, 437–442.

(21) Yagasaki, T.; Ono, J.; Saito, S. Ultrafast energy relaxation and anisotropy decay of the librational motion in liquid water: A molecular dynamics study. *J. Chem. Phys.* **2009**, *131*, 164511.

(22) Schmidt, J. R.; Corcelli, S. A.; Skinner, J. L. Pronounced Non-Condon Effects in the Ultrafast Infrared Spectroscopy of Water. *J. Chem. Phys.* **2005**, *123*, 044513.

(23) Tokmakoff, A. Orientational correlation functions and polarization selectivity for nonlinear spectroscopy of isotropic media. I. Third order. *J. Chem. Phys.* **1996**, *105*, 1–12.

(24) Rezus, Y. L. A.; Bakker, H. J. Orientational dynamics of isotopically diluted H2O and D2O. *J. Chem. Phys.* **2006**, *125*, 144512.

(25) Bakker, H.; Skinner, J. L. Vibrational Spectroscopy as a Probe of Structure and Dynamics in Liquid Water. *Chem. Rev.* **2010**, *110*, 1498.

(26) Geiger, A.; Mausbach, P.; Schnitker, J.; Blumberg, R. L.; Stanley, H. E. Structure and Dynamics of the Hydrogen Bond Network in Water by Computer Simulations. *J. Phys. (Paris)* **1984**, *45*, C7–13–C7–30.

(27) Lin, Y. S.; Pieniazek, P. A.; Yang, M.; Skinner, J. L. On the Calculation of Rotational

Anisotropy Decay, as Measured by Ultrafast Polarization-Resolved Vibrational Pump-Probe Experiments. *J. Chem. Phys.* **2010**, *132*, 174505.

(28) Laasonen, K.; Sprik, M.; Parrinello, M.; Car, R. "Ab initio" liquid water. *J. Chem. Phys.* **1993**, *99*, 9080–9089.

(29) Marx, D.; Hutter, J. Ab Initio Molecular Dynamics: Theory and Implemention. *Modern Methods and Algorithms of Quantum Chemistry, J.Grotendorst (Ed.) John von Neumann Institute for Computing, Jülich, NIC Series* **2000**, *1*, 301–499.

(30) Khatib, R.; Backus, E. H. G.; Bonn, M.; Perez-Haro, M.; Gaigeot, M.-P.; Sulpizi, M. Water Orientation and Hydrogen-Bond Structure at the Fluorite/Water Interface. *Sci. Rep.* **2016**, *6*, 24287.

(31) Khatib, R.; Hasegawa, T.; Sulpizi, M.; Backus, E. H. G.; Bonn, M.; Nagata, Y. Molecular Dynamics Simulations of SFG Librational Modes Spectra of Water at the Water-Air Interface. *J. Phys. Chem. C* **2016**, *120*, 18665–18673.

(32) Khatib, R.; Sulpizi, M. Sum Frequency Generation Spectra from Velocity-Velocity Correlation Functions. *J. Phys. Chem. Lett.* **2017**, *8*, 1310–1314.

(33) Goedecker, S.; Teter, M.; Hutter, J. Separable Dual-Space Gaussian Pseudopotentials. *Phys. Rev. B* **1996**, *54*, 1703–1710.

(34) Hartwigsen, C.; Goedecker, S.; Hutter, J. Relativistic separable dual-space Gaussian pseudopotentials from H to Rn. *Phys. Rev. B* **1998**, *58*, 3641–3662.

(35) Krack, M. Pseudopotentials for H to Kr optimized for gradient-corrected exchange-correlation functionals. *Theor. Chem. Acc.* **2005**, *114*, 145–152.

(36) Becke, A. D. Density-Functional Exchange-Energy Approximation with Correct Asymptotic Behavior. *Phys. Rev. A* **1988**, *38*, 3098.

(37) Lee, C.; Yang, W.; Parr, R. G. Development of the Colic-Salvetti Correlation-Energy Formula into a Functional of the Electron Density. *Phys. Rev. B* **1988**, *37*, 785.

(38) Grimme, S.; Antony, J.; Ehrlich, S.; Krieg, H. A Consistent and Accurate Ab Initio Parametrization of Density Functional Dispersion Correction (DFT-D) for the 94 Elements H-Pu. *J. Chem. Phys.* **2010**, *132*, 154104.

(39) Klimeš, J.; Michaelides, A. Perspective: Advances and Challenges in Treating van der Waals Dispersion Forces in Density Functional Theory. *J. Chem. Phys.* **2012**, *137*, 120901–120912.

(40) Lu, J.-B.; Cantu, D. C.; Nguyen, M.-T.; Li, J.; Glezakou, V.-A.; Rousseau, R. Norm-Conserving Pseudopotentials and Basis Sets To Explore Lanthanide Chemistry in Complex Environments. *J. Chem. Theory Comput.* **2019**, *15*, 5987–5997.

(41) VandeVondele, J.; Krack, M.; Mohamed, F.; Parrinello, M.; Chassaing, T.; Hutter, J. Quickstep: Fast and Accurate Density Functional Calculations using a Mixed Gaussian and Plane Waves Approach. *Comput. Phys. Commun.* **2005**, *167*, 103–128.

(42) Kühne, T. D.; Iannuzzi, M.; Del Ben, M.; Rybkin, V. V.; Seewald, P.; Stein, F.; Laino, T.; Khaliullin, R. Z.; Schütt, O.; Schiffmann, F.; Golze, D.; Wilhelm, J.; Chulkov, S.; Bani-Hashemian, M. H.; Weber, V.; Borštnik, U.; Taillefumier, M.; Jakobovits, A. S.; Lazzaro, A.; Pabst, H.; Müller, T.; Schade, R.; Guidon, M.; Andermatt, S.; Holmberg, N.; Schenter, G. K.; Hehn, A.; Bussy, A.; Belleflamme, F.; Tabacchi, G.; Glöß, A.; Lass, M.; Bethune, I.; Mundy, C. J.; Plessl, C.; Watkins, M.; VandeVondele, J.; Krack, M.; Hutter, J. CP2K: An electronic structure and molecular dynamics software package - Quickstep: Efficient and accurate electronic structure calculations. *J. Chem. Phys.* **2020**, *152*, 194103.

(43) Lippert, G.; Hutter, J.; Parrinello, M. The Gaussian and Augmented-Plane-Wave Den-

sity Functional Method for Ab Initio Molecular Dynamics Simulations. *Theor. Chem. Acc.* **1999**, *103*, 124.

(44) Martyna, G. J.; Klein, M. L.; Tuckerman, M. Nosé-Hoover Chains: The Canonical Ensemble via Continuous Dynamics. *J. Chem. Phys.* **1992**, *97*, 2635.

(45) Babin, V.; Leforestier, C.; Paesani, F. Development of a "First Principles" Water Potential with Flexible Monomers: Dimer Potential Energy Surface, VRT Spectrum, and Second Virial Coefficient. *J. Chem. Theory Comput.* **2013**, *9*, 5395–5403.

(46) Babin, V.; Medders, G. R.; Paesani, F. Development of a "First Principles" Water Potential with Flexible Monomers. II: Trimer Potential Energy Surface, Third Virial Coefficient, and Small Clusters. *J. Chem. Theory Comput.* **2014**, *10*, 1599–1607.

(47) Medders, G. R.; Babin, V.; Paesani, F. Development of a "First-Principles" Water Potential with Flexible Monomers. III. Liquid Phase Properties. *J. Chem. Theory Comput.* **2014**, *10*, 2906–2910.

(48) Bore, S. L.; Paesani, F. Realistic phase diagram of water from "first principles" data-driven quantum simulations. *Nat. Commun.* **2023**, *14*.

(49) Thompson, A. P.; Aktulga, H. M.; Berger, R.; Bolintineanu, D. S.; Brown, W. M.; Crozier, P. S.; in 't Veld, P. J.; Kohlmeyer, A.; Moore, S. G.; Nguyen, T. D.; Shan, R.; Stevens, M. J.; Tranchida, J.; Trott, C.; Plimpton, S. J. LAMMPS - a flexible simulation tool for particle-based materials modeling at the atomic, meso, and continuum scales. *Comput. Phys. Commun.* **2022**, *271*, 108171.

(50) Alejandre, J.; Tildesley, D. J.; Chapela, G. A. Molecular dynamics simulation of the orthobaric densities and surface tension of water. *J. Chem. Phys.* **1995**, *102*, 4574–4583.

(51) Reddy, S. K.; Straight, S. C.; Bajaj, P.; Huy Pham, C.; Riera, M.; Moberg, D. R.; Morales, M. A.; Knight, C.; Götz, A. W.; Paesani, F. On the accuracy of the MB-

pol many-body potential for water: Interaction energies, vibrational frequencies, and classical thermodynamic and dynamical properties from clusters to liquid water and ice. *J. Chem. Phys.* **2016**, *145*, 194504.

(52) Muniz, M. C.; Gartner, I., Thomas E.; Riera, M.; Knight, C.; Yue, S.; Paesani, F.; Panagiotopoulos, A. Z. Vapor–liquid equilibrium of water with the MB-pol many-body potential. *J. Chem. Phys.* **2021**, *154*, 211103.

(53) Vargaftik, N. B.; Volkov, B. N.; Voljak, L. D. International Tables of the Surface Tension of Water. *J. Phys. Chem. Ref. Data.* **1983**, *12*, 817–820.

(54) Nagata, Y.; Ohto, T.; Bonn, M.; Kühne, T. D. Surface tension of ab initio liquid water at the water-air interface. *J. Chem. Phys.* **2016**, *144*, 204705.

(55) Muller, E. A.; Ervik, ; Mejía, A. A Guide to Computing Interfacial Properties of Fluids from Molecular Simulations [Article v1.0]. *LiveCoMS* **2020**, *2*.

(56) Tang, F.; Ohto, T.; Hasegawa, T.; Xie, W. J.; Xu, L.; Bonn, M.; Nagata, Y. Definition of Free O–H Groups of Water at the Air–Water Interface. *J. Chem. Theory Comput.* **2017**, *14*, 357–364.
